# Effects of bovine leukemia virus infection on milk neutrophil function and the milk lymphocyte profile

**DOI:** 10.1186/s13567-014-0125-4

**Published:** 2015-01-17

**Authors:** Alice Maria Melville Paiva Della Libera, Fernando Nogueira de Souza, Camila Freitas Batista, Bruna Parapinski Santos, Luis Fernando Fernandes de Azevedo, Eduardo Milton Ramos Sanchez, Soraia Araújo Diniz, Marcos Xavier Silva, João Paulo Haddad, Maiara Garcia Blagitz

**Affiliations:** Departamento de Clínica Médica, Faculdade de Medicina Veterinária e Zootecnia, Universidade de São Paulo, Av. Prof. Dr. Orlando Marques de Paiva, 87, Cidade Universitária, São Paulo, 05508-270 Brazil; Departamento de Medicina Veterinária Preventiva, Escola de Veterinária, Universidade Federal de Minas Gerais, Belo Horizonte, 31270-010 Brazil; Laboratório de Sorologia e Imunobiologia, Instituto de Medicina Tropical, Universidade de São Paulo, São Paulo, 05403-000 Brazil

## Abstract

The effects of bovine leukemia virus (BLV) on the immune response have been extensively investigated; however, its effects on mammary gland immunity are only speculative. Although BLV has a tropism for B cells, it can affect both adaptive and innate immunities because these systems share many effector mechanisms. This scenario is the basis of this investigation of the effects of BLV on mammary gland immunity, which is largely dependent upon neutrophilic functions. Thus, the present study sought to examine neutrophilic functions and the lymphocyte profile in the milk of naturally BLV-infected cows. The viability of the milk neutrophils and the percentage of milk neutrophils that produced reactive oxygen species (ROS) or phagocytosed *Staphylococcus aureus* were similar between BLV-infected and BLV-uninfected dairy cows. Furthermore, the expression of CD62L and CD11b by the milk neutrophils and the percentage of milk neutrophils (CH138^+^ cells) that were obtained from the udder quarters of the BLV-infected cows were not altered. Conversely, the median fluorescence intensity (MFI) representing intracellular ROS production and the phagocytosis of *S. aureus*, the expression of CD44 by the milk neutrophils and the percentage of apoptotic B cells were lower in the milk cells from BLV-infected dairy cows, particularly those from animals with persistent lymphocytosis (PL). The lymphocyte subsets were not different among the groups, with the exception of the percentage of CD5^−^/CD11b^−^ B cells, which was higher in the milk cells from BLV-infected cows, particularly those with PL. Thus, the present study provides novel insight into the implications of BLV infection for mammary gland immunity.

## Introduction

Bovine leukemia virus (BLV) is a member of the Retroviridae family and the *Deltaretrovirus* genus that is genetically and structurally similar to the primate T-lymphotropic viruses types 1–5 (i.e., HTLV-1 to 4). Although BLV has been successfully eradicated in some regions of Europe, it is among the most widespread livestock pathogens in many countries, particularly in dairy herds. BLV infections in cattle may remain clinically silent or present as persistent lymphocytosis (PL); more rarely, BLV infection may result in B cell lymphoma. PL is characterized by a chronic elevation in the number of circulating B lymphocytes and is found in approximately 20-30% of BLV-infected cattle [1].

Various studies have investigated the effects of BLV infection on lymphocyte subsets [1–8], neutrophil functions [9–14] and B cell viability [15–22]. All of these studies evaluated these parameters in the blood, and therefore, the effects of BLV infection on the lymphocyte subsets, neutrophil functions and B cell viability in milk are only speculative. However, mastitis [23–30] and decreased milk production [31–35] have been associated with BLV infection, particularly in BLV-infected cows with PL [31] and high-performing infected dairy herds [35]. These findings prompted an investigation into the effects of BLV, which is a B cell tropic virus [1,6,15], on mammary gland immunity, which is largely dependent upon neutrophil functions and recruitment. Notably, the impact of some chronic diseases with low lethality, such as BLV infection, may be underestimated because they may be associated with comorbidities, such as mastitis, which is the most costly dairy cattle disease. This disease threatens the image of the dairy sector because of animal welfare issues and issues related to milk quality and public health due to increased risks of antimicrobial residues and the emergence of resistant bacteria. A better understanding of the implications of BLV infection on mammary gland immunity is critical for controlling mastitis and facilitating the strict control of these infections to improve dairy cattle productivity. Thus, the present study sought to explore milk lymphocyte subsets, neutrophil functions and B cell viability from naturally BLV-infected cows.

## Materials and methods

### Experimental design and collection of samples

The present study used 57 quarters of the mammary glands of 19 dairy cows in a commercial herd, at different lactation stages. Due to the effects of bacterial mastitis pathogens on neutrophil function [36–40], the following exclusion criteria were applied: 1) bacteriologically positive quarters; 2) quarters with abnormal secretions in the strip cup test; and 3) quarters with high somatic cell counts (SCCs) based on the previously proposed threshold for SCC [41,42].

The sera of all of the animals were tested for BLV using an agar gel immunodiffusion (AGID) assay (Tecpar®, Curitiba, Brazil) and an ELISA (VMRD Pullman Inc., Pullman, WA, USA, cat. number 284–5), using gp51 as the antigen. These animals were divided uniformly into the following three groups according to the sera test results: negative for BLV infection according to the AGID and ELISA assays and lacking hematological alterations [43] (healthy; *n* = 8; 24 quarters); positive for BLV according to both tests and lacking hematological alterations [43], which is commonly referred to as aleukemic (AL; *n* = 6; 16 quarters); and positive for BLV according to both tests and exhibiting PL (*n* = 5; 17 quarters). The BLV-infected cattle were classified as having PL when their lymphocyte counts exceeded 1 × 10^4^ mL^−1^ and their leukocyte counts exceeded 1.5 × 10^4^ mL^−1^ as established by Thurmond et al. [44]. One hundred ten days after the first sampling for the serodiagnosis of BLV, additional blood samples for the hematological procedures and serodiagnoses of BLV were collected to confirm the persistence of lymphocytosis. At this time (110 days after the first sampling), milk cells for the SCC, bacteriological analysis and flow cytometry analysis to determine neutrophil function and lymphocyte profile were also collected.

First, the strip cup test was performed to determine the presence of clots or flakes or otherwise obviously abnormal secretions. Then, predipping was performed, using one towel for each teat. After discarding the first three milk streams, the ends of the teats were scrubbed with 70% ethanol using a piece of cotton, and single milk samples from the individual mammary quarters were aseptically collected in sterile vials for the bacteriological analysis. Finally, milk samples for the SCC and the evaluation of neutrophilic function and lymphocyte profile were collected. The samples were maintained at 4 °C until they arrived at the laboratory. The milk samples for the bacteriological analysis were stored at −20 °C for a maximum of 30 days until the analysis.

Subsequently, each sample was codified and randomized, and further analyses were performed in which the researcher was blinded to the BLV status of the animal from which the sample was drawn. This research complied with the Ethical Principles for Animal Research and was approved by the Bioethics Commission.

### Hematological procedures

The total leukocyte counts were determined using an automated cell counter (ABX VET ABC, Horiba ABX Diagnostic®, Montpellier, France). The differential leukocyte counts were performed using routine smears.

### Bacteriological analysis

The bacteriological analysis was performed by culturing 0.01 mL of each milk sample on 5% sheep blood agar plates. The plates were incubated for 72 h at 37 °C, followed by Gram staining, observation of colony morphologies and biochemical testing [45]. A milk sample was considered to be culture-positive when the growth of ≥ 3 colonies was detected, with the exception of animals with *Staphylococcus aureus* or *Streptococcus agalactiae* infections in their quarters, which were considered to be culture-positive when the growth of ≥ 1 colony was detected [46,47].

### Determination of SCC

The milk samples for SCC determination were collected in 40-mL vials containing microtablets of the preservant agent bronopol (2-bromo-2-nitropane-1,3-diol). Subsequently, the SCC were performed using the Somacount 300 automated somatic cell counter (Bentley Instruments, Chaska, MN, USA).

### Separation of milk cells

The separation of the milk cells was performed as described by Koess and Hamann [48]. Briefly, 1 L of milk was diluted with 1 L of phosphate-buffered saline (PBS; pH 7.4; 1.06 mM Na_2_HPO_4_, 155.17 mM NaCl and 2.97 mM Na_2_HPO_4_.7H_2_O). After centrifugation at 1000 × *g* for 15 min, the cream layer and supernatant were discarded. The cell pellet was then washed once using 30 mL of PBS and centrifuged at 400 × *g* for 10 min. The cells were resuspended in 1 mL of RPMI-1640 nutritional medium (R7638, Sigma Aldrich, USA) supplemented with 10% fetal bovine serum (Cultilab, Brazil) and counted using a Neubauer chamber. Cell viability was first evaluated by trypan blue exclusion. The milk cells were then diluted with nutritional medium containing 10% fetal bovine serum to a concentration of 2 × 10^6^ viable cells mL^−1^.

### Enumeration of lymphocyte subpopulations

The cells were washed with PBS and stained to detect the combination of CD3, CD4 and CD8, the combination of CD3, CD4 and CD25 and the combination of CD21, CD5 and CD11b following incubation with the primary antibodies (Abs) for 30 min at room temperature. The identification of lymphocyte subsets was based on their cytoplasmic granularities and mean fluorescence intensities following a two-step fluorescent immunolabeling protocol using primary anti-bovine monoclonal Abs and secondary Abs coupled to long-wavelength fluorescent probes. Thus, the following primary monoclonal antibodies (mAbs) directed against bovine lymphocytes were used: mouse IgG1 anti-bovine CD3 (T lymphocytes, MM1A, VMRD Pullman Inc. Corp®), mouse IgG2a anti-bovine CD4 (IL-A11, VMRD Pullman Inc. Corp®), mouse IgM anti-bovine CD8α (BAQ111A, VMRD Pullman Inc. Corp®), mouse IgG3 anti-bovine CD25 (LCTB2A; VMRD Pullman Inc. Corp®), mouse IgM anti-bovine CD21 (B lymphocytes, BAQ15A, VMRD Pullman Inc. Corp®), mouse IgG2a anti-bovine CD5 (B29A, VMRD Pullman Inc. Corp®) and mouse IgG1 anti-bovine CD11b (MM12A, VMRD Pullman Inc. Corp®). After washing with PBS, the cells were incubated for 30 min at room temperature with the following secondary Abs: goat anti-mouse IgG1 conjugated to phycoerythrin-Cy5 (PE-Cy5) (M32018; Invitrogen, Carlsbad, CA, USA), goat anti-mouse IgM conjugated to fluorescein isothiocyanate (FITC) (M31501, Invitrogen), goat anti-mouse IgG2a conjugated to phycoerythrin (PE) (M32204, Invitrogen) and goat anti-mouse IgG3 conjugated to FITC (M32701, Invitrogen). The cells were then washed with PBS and immediately analyzed using flow cytometry. A total of 20 000 milk cells, excluding most of the cellular debris, was examined per sample. The FlowJo software (TreeStar Inc., Ashland, OR, USA) was used to analyze the data. The results were corrected for autofluorescence content, which was defined as the fluorescence that was associated with the non-labeled freshly isolated milk cells from the same cow.

### Identification of neutrophils

Milk neutrophils were differentiated from other cells by indirect fluorescent labeling. The cells were incubated with an unlabeled primary monoclonal anti-bovine granulocyte antibody (anti-CH138A, VMRD Pullman Inc. Corp®) for 30 min at room temperature. Next, 1 mL of PBS was added to the cell suspension, which was centrifuged at 400 × *g* for 8 min. Finally, a labeled secondary Ab was added, and the sample was incubated for 30 min at room temperature in the dark to visualize the bound CH138A. The neutrophils were identified using flow cytometry based on the cells’ cytoplasmic granularities and CH138A positivities as previously described [40,49]. The labeled secondary mAbs included allophycocyanin- (APC; M31505, Invitrogen), FITC- (M31501, Invitrogen) or PE-conjugated (M31504, Invitrogen) goat anti-mouse IgM mAb. A total of 20 000 milk cells, excluding most of the cellular debris, was examined per sample. The FlowJo software (TreeStar Inc., Ashland, OR, USA) was used to analyze the data. The results were corrected for autofluorescence content, which was defined as the fluorescence that was associated with the non-labeled freshly isolated milk cells from the same cow.

### Detection of apoptosis by flow cytometry

The death of neutrophils (CH138^+^) and B cells (CD21^+^) was assessed using dual labeling with an annexin V antibody and propidium iodide (PI; K2350, APOPTEST-FITC, Dako Cytomation, The Netherlands) and flow cytometric analyses as previously described [40,49]. Briefly, 2 × 10^5^ viable milk cells were suspended in 100 μL of binding buffer (10 mM HEPES, 150 mM NaCl, 1 mM MgCl_2_ and 1.8 mM CaCl_2_) containing anti-annexin V-FITC antibody and incubated at room temperature for 20 min in the dark. Immediately before flow cytometry analysis, 5 μL of a 250 μg/mL PI solution was added. Next, the neutrophils were labeled using mAbs as described above.

To analyze the data, scatter plots were generated for the gated neutrophils or B cells. The living, nonapoptotic cells were negative for both FITC-labeled anti-annexin V and PI. The cells that were positive for FITC-labeled anti-annexin V but negative for PI were classified as apoptotic cells [40,49]. The necrotic subpopulation was excluded from the analysis [49]. A total of 20 000 milk cells, excluding most of the cellular debris, was examined per sample. The FlowJo software (TreeStar Inc., Ashland, OR, USA) was used to analyze the data.

### Intracellular reactive oxygen species production

Intracellular reactive oxygen species (ROS) production was assessed by flow cytometry using 2′,7′-dichlorofluorescein diacetate (DCFH-DA) as a probe [50]. Briefly, 2 × 10^5^ viable milk cells from each quarter that were previously assessed by trypan blue exclusion were incubated at 37 °C for 30 min with 0.3 μM DCFH-DA (D6883, Sigma Aldrich, St. Louis, MO, USA).

The intracellular 2′,7′-dichlorofluorescein (DCF) fluorescence of the neutrophils was determined by flow cytometry using an excitation wavelength of 488 nm. DCFH-DA, which is a cell-permeable, nonfluorescent probe, is converted to DCF by ROS in a dose-dependent manner, resulting in fluorescence emission. The green fluorescence of DCF was detected at 500–530 nm.

The percentage of neutrophils producing ROS was calculated as the number of fluorescent neutrophils divided by the total neutrophil count and multiplied by 100. The median fluorescence intensity (MFI) of ROS production was estimated from the median of DCF fluorescence divided by the number of neutrophil that produced ROS [40]. For this assay, 10 000 gated neutrophils were examined per sample. The FlowJo software (TreeStar Inc., Ashland, USA) was used to analyze the data. The results were corrected for autofluorescence content, which was defined as the fluorescence that was associated with the non-labeled freshly isolated milk cells from the same cow.

### Preparation of PI-labeled bacteria

PI-labeled *Staphylococcus aureus* (ATCC 25923) was prepared as proposed by Hasui et al. [50] with some modifications. Briefly, *S. aureus* was cultured for 18 h at 37 °C on brain-heart infusion agar. Subsequently, the bacteria were heat-killed by incubation at 60 °C for 30 min, after which they were washed three times using a sterile saline solution (0.9% NaCl). The bacterial density was adjusted to an absorbance of 2.50 at 620 nm, yielding approximately 2.4 × 10^9^ bacteria mL^−1^, as previously described [50]. The bacteria were then labeled using a 5% PI (P4170, Sigma Aldrich, St. Louis, MO, USA) solution for 30 min at room temperature. The fluorescent bacteria were washed three times and suspended in PBS containing 5 mM glucose and 0.1% gelatin, and aliquots were stored at −80 °C. Thereafter, the PI labeling of the bacteria was confirmed by flow cytometry.

### Phagocytosis assay

The phagocytosis assay was performed using flow cytometry of PI-labeled *S. aureus* as previously described by Hasui et al. [50]. Briefly, 2 × 10^5^ viable milk cells were incubated with 100 μL of heat-killed, PI-labeled *S. aureus* and 900 μL of PBS for 30 min at 37 °C. Subsequently, 2 mL of 3 mM EDTA was added, and after centrifugation at 400 × *g* for 10 min, the leukocytes were resuspended in 300 μL of PBS and analyzed by flow cytometry.

The percentage of neutrophils that phagocytized the bacteria was equal to the number of fluorescent neutrophils divided by the total neutrophil count and multiplied by 100. The MFI of *S. aureus* phagocytosis was estimated from the median value of PI fluorescence divided by the number of neutrophils that phagocytized *S. aureus* [40]. At least 20 000 cells were examined per sample. The Flow Jo Tree Star Software (TreeStar Inc., Ashland, OR, USA) was used to analyze the data.

### Expression of L-selectin, β_2_-integrin and CD44

The identification of neutrophils expressing L-selectin (CD62L), the β-chain of β_2_-integrin (CD11b) and one of the three endothelial-selectin (E-selectin) ligands (CD44) was performed by flow cytometry using the following mAbs: a FITC-conjugated mouse anti-bovine CD62L (MCA1649F, AbDSerotec, Oxford, England), a primary mouse IgG1 anti-CD11b mAb (MM12A, Pullman Inc. Corp®), a phycoerythrin-Cy5 (PE-Cy5)-conjugated goat anti-mouse IgG1 Ab (M32018, Invitrogen), a primary mouse IgG3 anti-CD44 mAb (BAG40A, Pullman Inc. Corp®) and an FITC-conjugated goat anti-mouse IgG3 Ab (M32701, Invitrogen). First, dot plots of gated neutrophils (CH138A^+^) were generated as previously described. The neutrophils were identified as previously described. Then, unlabeled primary mAbs that were directed against CD11b and CD44 were added to the cell suspension and incubated for 30 min at room temperature. The isolated milk cell suspension was centrifuged at 400 × *g* for 8 min, and a labeled CD62L mAb and secondary labeled mAbs for the detection of the anti-CD11b and -CD44 Abs were added. Finally, the isolated milk cells were incubated for 30 min at room temperature in the dark to allow for the visualization of cells expressing CD62L, CD11b and CD44. We chose the relative MFI because this parameter was much more discriminating compared with the percentage of positive cells. The MFI provides an accurate measurement of the brightness of the stained cells and is thus an indicator of the number of receptors per cell [51]. For this assay, 10 000 gated neutrophil cells were examined per sample. The Flow Jo Tree Star Software (TreeStar Inc., Ashland, OR, USA) was used to analyze the data.

### Statistical analyses

First, the distributions of all of the variables were examined using normal probability plots obtained using the Shapiro and Wilk tests. The data were analyzed using a multivariate analysis of variance. Then, the Kruskal-Wallis and Mann–Whitney tests were applied. The model considered the quarters and the cows to be nested within the cows. The statistical analyses were performed using the STATA statistical software version 12 (Stata Corp., College Station, Texas, USA). The results are reported as the mean ± standard deviation. Significance was set at *P ≤* 0.05.

## Results

The results are summarized in Tables 1, 2 and 3. The SCC, lactational status and parity (data not shown) values did not differ among the groups. Here, we found that BLV infection detrimentally affected some important milk neutrophilic functions. For instance, the MFI that represented the amount of intracellular ROS production by the milk neutrophils was lower for the BLV-infected cows, particularly those with PL, than for the uninfected cows. Furthermore, the MFI that represented the amount of *S. aureus* phagocytosis by the milk neutrophils was also the lowest for the PL group. Moreover, the level of CD44 expression by milk neutrophils from the BLV-infected dairy cows was lower than that of milk neutrophils collected from the uninfected cows (Table 1).

**Table 1:** **Characteristics of neutrophils in the milk of healthy and bovine leukemia virus (BLV)-infected cows**

**Group/Variable**	**Negative (** ***n*** **= 24)**	**AL (** ***n*** **= 16)**	**PL (** ***n*** **= 17)**
**CH138** ^**+**^ **(%)**	13.72 ± 14.91^a^	8.89 ± 9.72^a^	11.88 ± 15.34^a^
**Annexin V** ^**−**^ **/PI** ^**−**^ **(%)**	30.74 ± 12.92^a^	39.05 ± 16.06^a^	24.97 ± 16.01^a^
**Annexin V** ^**+**^ **/PI** ^**−**^ **(%)**	39.50 ± 14.48^a^	37.03 ± 21.92^a^	39.27 ± 19.64^a^
**ROS production (%)**	54.91 ± 22.92^a^	68.20 ± 21.85^a^	69.98 ± 15.39^a^
**Intensity of ROS production (MFI)**	2069 ± 1008^a^	1603 ± 585.7^b^	865.6 ± 447.3^c^
***S. aureus*** **phagocytosis (%)**	63.23 ± 17.80^a^	55.11 ± 20.88^a^	58.84 ± 15.21^a^
**Intensity of** ***S. aureus*** **phagocytosis (MFI)**	219.8 ± 100.3^a^	211.2 ± 80.77^a^	104.2 ± 39.11^b^
**CD44 expression (MFI)**	22.03 ± 22.65^a^	1.07 ± 0.18^b^	1.03 ± 0.09^b^
**CD62L expression (MFI)**	13.45 ± 15.41^a^	1.01 ± 0.00^a^	1.01 ± 0.00^a^
**CD11b expression (MFI)**	755.8 ± 344.6^a^	633.3 ± 555.0^a^	843.7 ± 334.9^a^

Different superscripted letters^a,b,c^ within a row indicate significant differences (*P ≤* 0.05) between the values. The results are shown as the mean ± SD.

AL: aleukemic BLV-infected cows; PL: BLV-infected cows with persistent lymphocytosis; PI: propidium iodide; *S. aureus*: *Staphylococcus aureus*; ROS: reactive oxygen species; MFI: median fluorescence intensity.

**Table 2:** **Percentage of lymphocyte subsets in the milk from healthy and bovine leukemia virus (BLV)-infected cows**

**Group/Variables**	**Negative (** ***n*** **= 25)**	**AL (** ***n*** **= 16)**	**PL (** ***n*** **= 17)**
**CD3** ^**+**^ **(T cells) (%)**	7.13 ± 5.04^a^	10.46 ± 9.30^a^	11.66 ± 7.27^a^
**CD4** ^**+**^ **/CD8** ^**−**^ **T cells (%)**	1.32 ± 1.19^a^	1.83 ± 1.73^a^	2.55 ± 2.48^a^
**CD4** ^**−**^ **/CD8** ^**+**^ **T cells (%)**	3.15 ± 2.57^a^	4.45 ± 2.02^a^	4.51 ± 3.86^a^
**CD4** ^**+**^ **/CD8** ^**+**^ **T cells (%)**	0.41 ± 0.36^a^	0.33 ± 0.30^a^	0.23 ± 0.19^a^
**CD4** ^**−**^ **/CD8** ^**−**^ **T cells (%)**	2..24 ± 2.33^a^	3.84 ± 5.74^a^	4.38 ± 3.87^a^
**CD3** ^**+**^ **(T cells) (%)**	6.31 ± 4.99^a^	10.28 ± 10.44^a^	11.06 ± 6.81^a^
**CD4** ^**+**^ **/CD25** ^**−**^ **T cells (%)**	1.38 ± 1.32^a^	1.84 ± 1.67^a^	2.70 ± 2.46^a^
**CD4** ^**−**^ **/CD25** ^**+**^ **T cells (%)**	0.06 ± 0.04^a^	0.13 ± 0.18^a^	0.15 ± 0.14^a^
**CD4** ^**+**^ **/CD25** ^**+**^ **T cells (%)**	0.24 ± 0.23^a^	0.21 ± 0.29^a^	0.29 ± 0.36^a^
**CD4** ^**−**^ **/CD25** ^**−**^ **T cells (%)**	4.63 ± 4.11^a^	8.11 ± 8.58^a^	7.92 ± 4.62^a^
**CD21** ^**+**^ **(B cells) (%)**	7.90 ± 5.53^a^	13.02 ± 8.14^a^	17.44 ± 7.20^a^
**CD5** ^**+**^ **/CD11** ^**−**^ **B cells (%)**	0.87 ± 0.88^a^	0.71 ± 0.45^a^	0.72 ± 0.47^a^
**CD5** ^**−**^ **/CD11** ^**+**^ **B cells (%)**	3.84 ± 4.01^a^	6.80 ± 6.97^a^	8.59 ± 5.15^a^
**CD5** ^**+**^ **/CD11** ^**+**^ **B cells (%)**	0.85 ± 0.78^a^	0.85 ± 0.51^a^	1.01 ± 0.66^a^
**CD5** ^**−**^ **/CD11** ^**−**^ **B cells (%)**	2.35 ± 1.73^a^	4.67 ± 3.26^b^	7.11 ± 3.89^c^

Different superscripted letters^a,b,c^ within a row indicate significant differences (*P ≤* 0.05) between the values. The results are shown as the mean ± SD.

AL: aleukemic BLV-infected cows; PL: BLV-infected cows with persistent lymphocytosis.

**Table 3:** **Viability of B cells in milk from healthy and bovine leukemia virus (BLV)-infected cows.**

**Group/Variables**	**Negative (** ***n*** **= 25)**	**AL (** ***n*** **= 16)**	**PL (** ***n*** **= 17)**
**Annexin V** ^**−**^ **/PI** ^**−**^ **(%)**	37.13 ± 17.99^a^	51.15 ± 16.73^a^	71.31 ± 13.02^a^
**Annexin V** ^**+**^ **/PI** ^**−**^ **(%)**	55.72 ± 17.59^a^	31.22 ± 18.43^b^	12.35 ± 6.44^c^

Different superscripted letters^a,b,c^ within a row indicate significant differences (*P* ≤ 0.05) between the values. The results are shown as the mean ± SD.

AL: aleukemic BLV-infected cows; PL: BLV-infected cows with persistent lymphocytosis; PI: propidium iodide.

The lymphocyte subsets did not differ among the groups, with the exception of the percentage of CD5^−^/CD11b^−^ B cells, which was higher in the BLV-infected cows, particularly those with PL (Table 2). Furthermore, the percentage of apoptotic B cells was lower in the BLV-infected dairy cows (Table 3), particularly those with PL, than in the uninfected cows.

## Discussion

It is not easy to precisely delineate innate immunity because it is intricately enmeshed with adaptive immunity, and the two systems share many effector mechanisms [37]. Thus, various viruses can affect the general functions of both innate and adaptive immunities. This phenomenon predisposes animals to different coinfections or superinfections and can increase the severity of infections [25,28,30,52–55]. As previously mentioned, the impact of some chronic diseases with low lethalities may be underestimated because of their associations with comorbidities. This scenario prompted an investigation into the effects of BLV, which is a B cell tropic virus [1,6,15], on mammary gland immunity, which is largely dependent on neutrophil function and recruitment [36–40].

Neutrophils form the first line of cellular defense against invading pathogens [36–40] and are essential for the innate host defense against invading microorganisms. These cells eliminate pathogens by a process known as phagocytosis. During phagocytosis, neutrophils produce ROS to kill invading pathogens [36–40,56].

Apoptosis of bovine neutrophils implies impaired phagocytic and oxidative burst activities [47,57,58]. Neutrophil viability is closely related to neutrophil phagocytosis and oxidative burst activities [56,58]. Thus, the non-significant difference that was observed in neutrophil viability rates among the groups in this study may be related to the results for the percentage of neutrophils that produced ROS or phagocytosed *S. aureus*.

While the study size is limited, we observed a lower MFI representing the phagocytosis of *S. aureus* and the intracellular production of ROS by the milk neutrophils from the BLV-infected cows, particularly those with PL. BLV-infected cows with PL have higher proviral loads [59,60] that are linked to lower levels of interferon (IFN)-γ expression by peripheral blood mononuclear cells [7,61–63], which is regulated by many factors, such as the PD-1 [7,62] and Tim-3/Gal-9 pathways [63]. IFN-γ has a positive effect on bovine neutrophil phagocytosis and ROS production [64], and this characteristic together with the altered production of IFN-γ by the peripheral blood mononuclear cells of BLV-infected cattle may explain our results regarding the deficient functions of the milk neutrophils. Consistent with our findings, Takamatsu et al. [11] found that most sera from leukemic cattle inhibit the phagocytosis of blood neutrophils.

In this study, no significant differences were observed in the levels of CD62L and CD11b expression by the milk neutrophils of BLV-infected cows. CD62L mediates the initial transient attachment of circulating granulocytes to the activated endothelium. The surface expression and rapid functional activation of Mac-1 (CD11b/CD18) is essential for the subsequent granulocyte migration to the site of inflammation. Following activation, CD62L is shed from the cell surface by proteolysis, whereas the surface expression of Mac-1 is up-regulated [51]. Therefore, the migration of neutrophils across endothelial cells is almost completely dependent upon CD18, the β-chain of β_2_ integrins and to a lesser extent on CD11b, which is one of the α-chains of β_2_ integrins [36].

Further, we found decreased levels of CD44 expression in the milk neutrophils from the BLV-infected dairy cows. CD44 was identified as one of the three endothelial-selectin ligands that are present on neutrophils, which are responsible for hindering their movement and activating their rolling. However, CD44 is required, but not essential, for neutrophil extravasation during inflammation [65]. CD44 is also regarded as a competent phagocytic receptor that efficiently mediates pathogen recognition and phagocytosis by neutrophils [66,67].

Together, these findings indicate that BLV infection, particularly BLV-infected cows with PL, may impact the outcome of intramammary infections because the resident milk neutrophils have an enormous impact on the elimination of bacteria by phagocytosis and the intracellular production of ROS [36,56]. Thus, we believe that BLV can affect mastitis control programs.

A feasible alternative to reduce the transmission of BLV infection could be achieved by eliminating animals with high proviral loads, which mainly exhibit PL instead of AL [59,60]. Thus, our findings regarding the milk neutrophil function in BLV-infected cows, particularly those with PL, indicated that the elimination of PL animals may also lead to a lower probability of comorbidities, such as mastitis, which is regarded as the most costly dairy cattle disease and is thus associated with important economic and public health implications.

No perturbations in the percentages of T lymphocyte subsets among the milk cells from the BLV-infected cows were found, as was previously described for the peripheral blood of BLV-infected cows [4,6], although no consensus exists [2,3,8]. It is generally accepted that the infected cells (mainly B cells) frequently co-express CD5 and CD11b molecules [1,6]. Conversely, the present study shows an increase in the percentage of CD5^−^/CD11b^−^ CD21^+^ cells (mainly B-1b cells) in the milk of BLV-infected cows, particularly those with PL. Although this observation is puzzling, it may lend insight into the roles of CD5 [68] and CD11b [69] on B cells in mammary gland immunity.

BLV infection is correlated with the inhibition of the apoptotic process, leading to the generation of a reservoir of apparently latent cells [1]. This phenomenon, together with the B cell tropism of BLV, may explain the lower percentage of milk B cells that was observed in the BLV-infected cows, particularly those with PL, that were undergoing apoptosis, which has been previously described for B cells that were obtained from blood [1,16,18,19,21,22].

In conclusion, the present study provides novel insight into the implications of BLV infections for mammary gland immunity, which is mainly supported here by the dysfunction of the milk neutrophils. Thus, this study highlights the importance of controlling BLV infections due to their indirect effects, such as the higher susceptibilities of BLV-infected cows to secondary diseases, such as mastitis, which is the most costly disease affecting cattle.

## Abbreviations

BLV: Bovine leukemia virus
ROS: Reactive oxygen species
MFI: Median fluorescence intensity
PL: Persistent lymphocytosis
SCC: Somatic cell count
AGID: Agar gel immunodiffusion
AL: Aleukemic
PBS: Phosphate-buffered saline
PE-Cy5: Phycoerythrin-Cy5
FITC: Fluorescein isothiocyanate
PE: Phycoerythrin
APC: Allophycocyanin
mAb: Monoclonal antibody
Ab: Antibody
PI: Propidium iodide
DCFH-DA: 2′,7′-dichlorofluorescein diacetate
DCFH: 2′,7′-dichlorofluorescein
CD62L: L-selectin
CD11b: β-chain of β_2_-integrin
IFN-γ: Interferon-γ
